# Comparing the effects of ketorolac and Paracetamol on postoperative pain relief after coronary artery bypass graft surgery. A randomized clinical trial

**DOI:** 10.1186/s13019-020-01125-y

**Published:** 2020-05-11

**Authors:** Fatemeh Javaherforooshzadeh, Hasan Abdalbeygi, Farahzad Janatmakan, Behnam Gholizadeh

**Affiliations:** 1 Department of Cardiac Anesthesia, Ahvaz Anesthesiology and Pain Research Center, Ahvaz Jundishapur University of Medical Sciences, Ahvaz, Iran; 2 Department of Anesthesia, Ahvaz Anesthesiology and Pain Research Center, Ahvaz Jundishapur University of Medical Sciences, Ahvaz, Iran; 3 Atherosclerosis Research Center, Ahvaz Jundishapur University of Medical Sciences, Ahvaz, Iran

**Keywords:** Ketorolac, Paracetamol, Postoperative analgesia, Visual analog score, Cardiac surgery

## Abstract

**Introduction:**

Pain management after coronary artery bypass graft (CABG) surgery remains challenging.

**Objective:**

This study aimed to compare the effects of Ketorolac and Paracetamol on postoperative CABG pain relief.

**Method:**

This double-blind randomized clinical trial study was conducted in Ahvaz, Iran, from September 2018–December 2019. Two consecutive groups of 60 patients undergoing elective on-pump coronary artery bypass graft surgery.

**Intervention:**

The patients were divided into 0.5 mg/kg of ketorolac mg/dl and 10 mg/kg of Paracetamol after surgery for pain management**. Primary outcomes** were: visual analog pain scale (VAS) at the time point immediately after extubation (baseline) and at 6, 12, 24 and 48 h and the total dose of morphine consumption. **Secondary outcomes** included the hemodynamic variables, weaning time, chest tube derange, in-hospital mortality and myocardial infarction.

Statistical analysis**:** The data were analyzed using SPSS version 22(SPSS, Chicago, IL). The Mann-Whitney U-test was used to compare demographic data, VAS scores, vital signs, and side effects. Repeated measurements were tested within groups using Friedman’s ANOVA and the Wilcoxon rank-sum test. Values were expressed as means ± standard deviations. Statistical significance was defined as a *p*-value < 0.05.

**Results:**

Compared with baseline scores, there were significant declines in VAS scores in both groups throughout the time sequence (P< 0.05). The statistical VAS score was slightly higher in the Paracetamol group at most time points, except for the time of 6 h. However, at 24 and 48 h, the VAS score in group Paracetamol was significantly higher than in group Ketorolac. There were no significant differences between groups about hemodynamic variables.

**Conclusion:**

The efficacy of ketorolac is comparable to that of Paracetamol in postoperative CABG pain relief.

**Trial registry:**

IRCT20150216021098N5. Registered at 2019-09-12.

## Introduction

Control of pain management after coronary artery bypass graft (CABG) surgery remains challenging. Incompetently controlled postoperative pain can increase catecholamine levels, triggering myocardial ischemia, stroke, and bleeding complications. Limiting patient mobility, poorly managed postoperative pain can increase the risk of deep vein thrombosis and pneumonia, in addition to harmful psychological consequences such as insomnia and demoralization [1, 2]. Postoperative analgesia after cardiac surgery most commonly involves the use of intravenous and oral opioids. Intravenous (IV) opioids, such as morphine, are the analgesics commonly used to provide postoperative pain relief after CABG surgery [3, 4]. However, adverse effects, such as drowsiness, respiratory depression, excessive sedation, biliary spasm, depression of gastrointestinal motility, nausea and vomiting, and, particularly in the elderly, confusion caused by opioids may delay patient recovery and rehabilitation [3, 5]. To limit these adverse effects without sacrificing adequate pain management, nonsteroidal anti-inflammatory drugs (NSAIDs), increasingly are being applied in the postoperative setting. Although NSAIDs have potential side effects (bleeding, gastrointestinal ulceration, renal dysfunction, and post-operative bone healing), several studies have noted low complication rates associated with their short-term use after CABG when administered to appropriately selected patients [6, 7].

Paracetamol is usually considered to be a frail inhibitor of the synthesis of prostaglandins (PGs). However, the in vivo effects of Paracetamol are alike to those of the discerning cyclooxygenase-2 (COX-2) inhibitors. It is the most commonly suggested pain-relieving for the treatment of acute pain [8]. Its benefit over NSAIDs is its lack of interfering with platelet functions. Moreover, it is safe to administer to patients with a history of peptic ulcers or asthma [9]. Its mechanism of action may involve a central inhibition of COX-2 [10, 11], inhibition of nitric oxide generation via a blockade of the N-methyl-D-aspartate (NMDA) receptor, and activation of the descending serotonergic pathway. Paracetamol can cross the blood-brain barrier, producing a central analgesic effect [12, 13].

Ketorolac has been used for postoperative analgesia in combination with opioids. Several studies have reported that ketorolac is as effective as morphine or meperidine for analgesia after some types of surgical procedures [14]. However, because many studies report significant side effects of ketorolac, including coagulopathy, gastrointestinal problems, and nephrotoxicity there is increasing interest in the use of other classes of non-opioid analgesics [15, 16]. It remains unknown whether NSAID utilization rates after CABG have changed since the boxed warning was issued, although some groups have reported their continued use to select cardiac surgery patients [17, 18]. Management of postoperative pain is a major concern for anesthetists in patients undergoing CABG surgery. Due to the fact that the purpose of studies is to find the methods of pain management with the highest efficiency and the least side effects, and also according to the characteristics mentioned for Ketorolac and Paracetamol, the aim of our study was to compare the analgesic effects of Ketorolac and Paracetamol to find the appropriate anesthetic agent for postoperative CABG pain. Therefore, this study aimed to compare the effects of Ketorolac and Paracetamol on postoperative CABG pain relief.

## Material and method

### Study design

This double-blind randomized clinical trial study was conducted in Golestan Hospital, Ahvaz, Iran, from September 2018–December 2019 with Ethics code: IR.AJUMS.REC.1398.050 from Anesthesiology and Pain Research Center, Ahvaz Jundishapur University of Medical Sciences, Ahvaz, Iran, and Trial registration number: IRCT20150216021098N5.

Sixty patients undergoing elective on-pump coronary artery bypass graft surgery. After clearly explaining the objective and potential risks and benefits of the study, a written consent form for participation in the study was obtained from all patients.

### Setting and patients: inclusion criteria

Aged 30–70 years, ASA III, Both of sex, Ejection Fraction≥30%, undergoing elective CABG.

### Exclusion criteria included

Severe hepatic and renal disease, consumption of anti-inflammatory drugs or antipyretic drugs before the study, redo surgery, history of cerebrovascular accident (CVA), and thrombocytopenia.

**Randomization**: randomization was performed using computer-generated random digits to ensure that patients and investigators were blind to the treatment assignment before study entry, and the allocation was done 1:1 to receive either ketorolac or Paracetamol. Randomization was not performed until electronically confirming the eligibility criteria in the web-based case report form. Randomization was performed centrally without stratification. The sequence was generated by an independent statistician using a random number generator with a 1:1 allocation using random block sizes of 2. In this study patients and researchers were blinded.

### Sample size

**Fig. 1:**
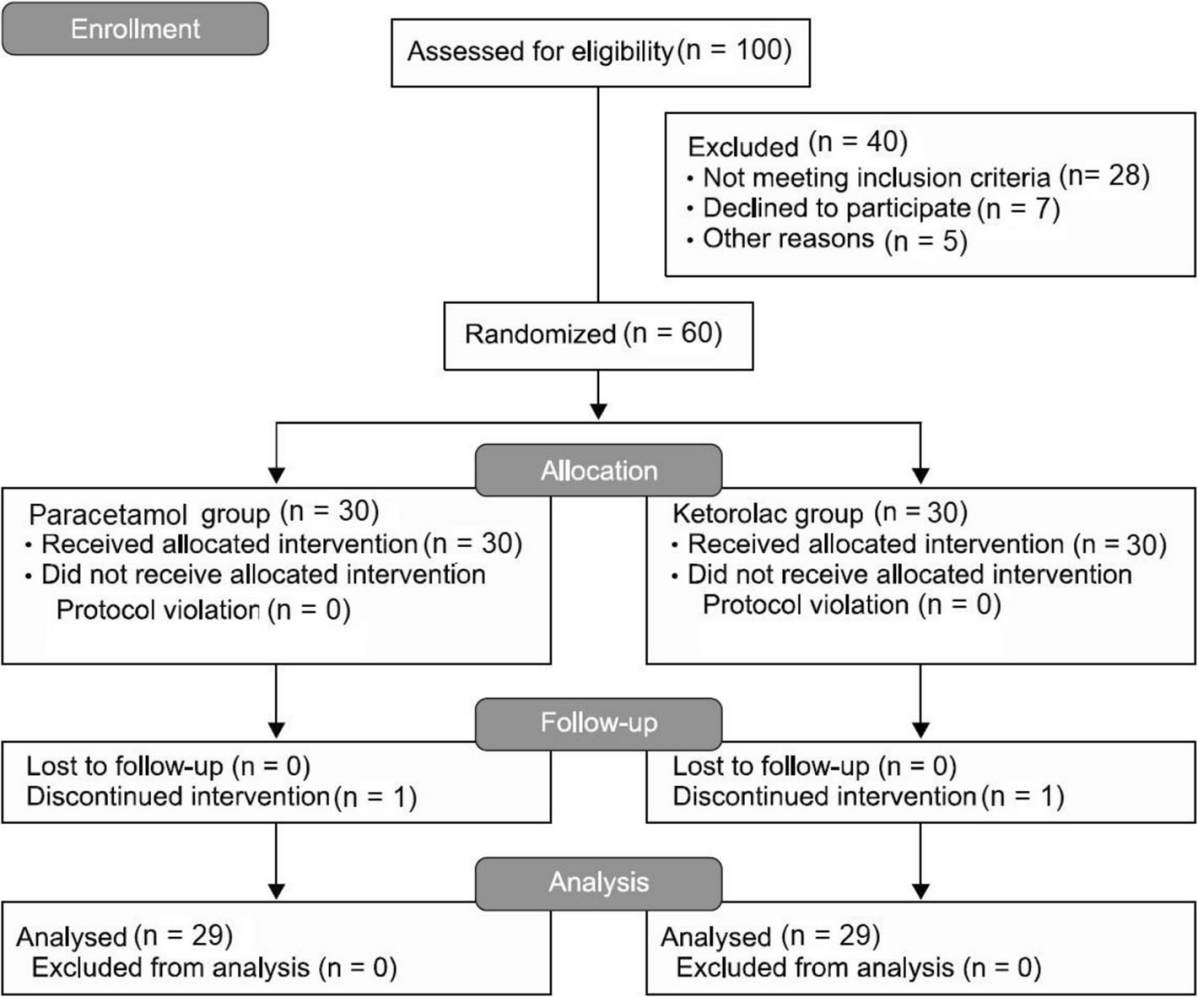
The consort flow chart

The sample size of this study was calculated using the sample size estimation formula. The 95% confidence interval (CI) level was considered. The study population consisted of 90 patients. Based on the previous data [19]. After initial screening, 85 patients agreed to participate and provided informed consent. Among them, 25 patients did not have inclusion criteria (Five patients had EF < 30%, Ten patients had complex surgery, the surgical procedure of Five patients turned into off-pump, Five patients were given antipsychotic drugs or history of seizure). Finally, 60 patients were enrolled in the study and were assigned into two groups of Paracetamol and ketorolac, 30 patients each (Fig. 1).

### Anesthesia protocol

After arrival to the operation room, standard monitoring included five-lead electrocardiography, pulse oximetry and arterial line for continuous blood pressure monitoring and blood gases were inserted. A standard anesthesia technique was used for all patients. The anesthetic drug doses were calculated according to body weight; midazolam at 0.1 mg/kg was given as IV premedication. Induction of anesthesia was induced fentanyl 15 μg/kg, propofol 1 mg/kg, and pancuronium 0.1–0.15 mg/kg). Anesthesia was maintained with a continues infusion of propofol 1–1.5 mg/kg/h, fentanyl 4 μg/kg/h, 0.25 mg/kg/h midazolam, and 0.3 mg/kg/h cisatracurium until the end of surgery. Isoflurane supplementation was used at the discretion of the attending anesthesiologist. After induction of general anesthesia, a central venous catheter was introduced. For initiation of cardiopulmonary bypass, 350 u/kg heparin was injected to all patients. Heparin dosage was attuned based on goal ACT 450–480 s. After the bypass was terminated, protamine 1 mg/kg was given for the reversal of heparin. Cardiac surgery and postoperative management were standardized.

After surgery, all the patients were admitted to the cardiovascular ICU, with a standard protocol for sedation, analgesia (propofol 0.5 mg/kg/h and morphine sulfate 0.1 mg/kg/h), and management of mechanical ventilation (SIMV mode of ventilation).

### Intervention

Immediately after the transfer of patients to ICU, the intervention began. The patients in the ketorolac group were administered at a rate of 0.5 mg/kg IV admixed with 100 ml of normal saline each 6 h for 24 h. A bolus may be given at the start of therapy if desired. The patients in the Paracetamol group were given 10 mg/kg IV of Paracetamol (mixed with normal saline to a total volume of 100 ml) for 30 min each 6 h for 24 h. (Dose of ketorolac determined based on Howard et.al study [20].

Patients were extubated according to the following criteria: responsive and cooperative, pO2 of 80–100, oxygenation index of pO2/FiO2 > 300, and hemodynamic stability without any inotropes.

After extubation, the patients had access to morphine sulfate with a patient-controlled analgesia device (PCA device; Graseby 3300P, Hoyer, Bremen, Germany) using a standardized protocol: bolus dose of 2 mg, dose duration of 2 min, lockout interval of 13 min (15 min effective lockout time), and with no background infusion and no upper dose limit. Before commencing the PCA, the nurses in the PACU were allowed to give morphine sulfate 2mgIV before extubation for the treatment of pain if required. Morphine consumption was recorded from the PCA device at the end of the 48-h study period. Per protocol, the primary efficacy variable was cumulative morphine consumption (the combined amount administered via the PCA device and given as rescue doses) at the end of the 48-h postoperative period. If pain relief was insufficient (VAS score > 3 at rest), nurses were allowed to give an extra bolus of morphine2 mg IV as a rescue analgesic once an hour. Renal function test (serum creatinine) were measured the evening before surgery and 72 h after surgery. Bleeding after surgery was measured as chest tube drainage. The number of red cell units transfused was recorded.

Primary outcomes were: visual analog pain scale (VAS) at the time point immediately after extubation (baseline) and at 6, 12, 24 and 48 h and the total dose of morphine consumption. Secondary outcomes included the hemodynamic variables, weaning time, postoperative bleeding, myocardial infarction, CVA, TIA, in-hospital mortality and postoperative serum creatinine.

### VAS score assessment

Pain intensity levels were subjectively measured using a 10 cm visual analog pain scale (VAS, 0 = no pain to 10 = unbearable pain). We assessed VAS and hemodynamic variables (systolic blood pressure, diastolic blood pressure, heart rate and other parameters) of each regimen immediately after extubation (baseline) and at 6, 12, 24, and 48 h.

### Statistical analysis

Numerical variables were reported as mean ± standard deviation (SD). Quantitative and qualitative variables were measured by independent t-test, and ANOVA test respectively. *P* value ≤0.05 was considered to be statistically significant. All analyses were performed using SPSS for Windows version 22.0 (SPSS Inc., Chicago, IL, USA).

## Results

During the study period from September 2018–December 2019, 100 patients undergoing elective on-pump CABG surgery were eligible to participate in the trial. Forty patients did not have inclusion criteria Finally, 60 patients were enrolled in the study and were assigned into two groups of ketorolac and Paracetamol, 30 patients each. (Fig. 1).

There were no significant differences between the two groups in terms of demographic characteristics including age, male/female ratio, antiplatelet using, Euro Score ‖ and duration of cross-clamp time (*P* > 0.05) (Table 1).

**Table 1:** Demographic Data of patients in Paracetamol and ketorolac groups

Parameter	Paracetamol (***n*** = 30)	Ketorolac group (***n*** = 30)	***P***-value
Male, n(%)	18 (60)	19 (63.33)	0.453
Age (year)	58.41 ± 1.82	61.83 ± 1.54	0.159
Weight (kg)	70.63 ± 10.10	74.80 ± 31.14	0.475
Height (cm)	173.03 ± 10.16	163.29 ± 27.20	0.063
Outpatient use of NSAIDs or antiplatelet, n (%)	20 (67)	21 (70)	0.21
Euro Score ‖ (%), mean ± SD	2.63 ± 2.65	2.86 ± 2.83	0.489
Duration of AO (min)	55 ± 38	49 ± 30	0.09

**Values are mean ± sd or number of patients.; Euro Score ‖: _ European System for Cardiac Operative Risk Evaluation;** ***AO*** **Aortic occlusion.*SD*** **Standard deviation**

**Fig. 2:**
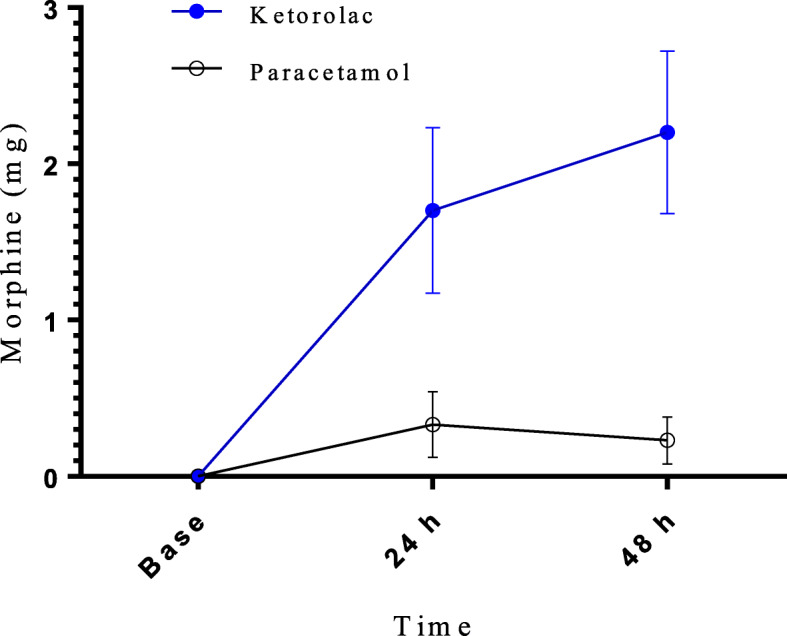
Postoperative morphine administration

There were significant differences about morphine consumption in two groups at 24 h (0.29 ± 0.41 mg in Paracetamol group versus 1.71 ± 0.53 mg in ketorolac group *p* = 0.027) and 48 h (0.22 ± 0.15 mg in Paracetamol versus 2.18 ± 0.52 mg in ketorolac group *p* = 0.007) after extubation (Fig. 2).

**Fig. 3:**
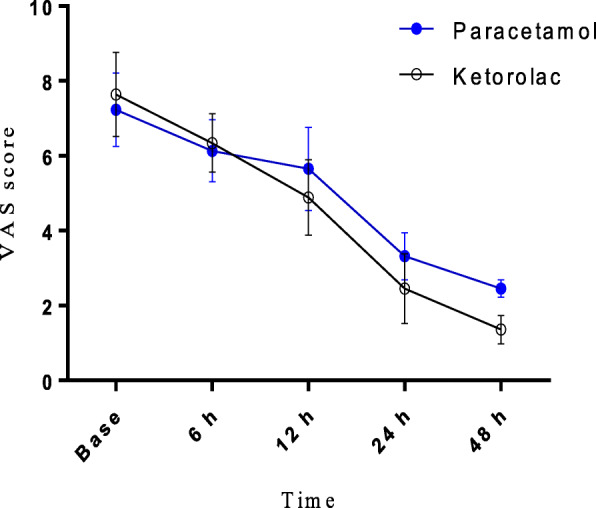
VAS score after the operation. There were significant VAS score declines in both groups (*P* < 0.05)

Compared with baseline scores, there were significant declines in VAS scores in both groups throughout the time sequence The statistical VAS score was higher in the Paracetamol group at most time points, except for the time of 6 h. However, at 24 and 48 h, the VAS score in group Paracetamol was significantly higher than in group Ketorolac (P< 0.05) (Fig. 3).

Comparison of the two groups receiving ketorolac and Paracetamol showed that there was no significant difference between the two groups in terms of platelet count, bleeding time, and chest tube derange Table 2. Hemodynamic parameters and SPO2 and postoperative bleeding at different times were not different between the two groups. Table 3.

**Table 2:** Effect of administration of ketorolac and Paracetamol on Bleeding Time, Platelet and post-operative bleeding

parameter	Paracetamol (***n*** = 30)	Ketorolac (***n*** = 30)	***p***-value
BT in 24 h. (S)	121.1 ± 2.73	126.8 ± 3.41	0.417
BT in 48 h. (S)	129.6 ± 34.56	132.4 ± 23.87	0.356
Decrease in platelet, median	74 (11–121)	72 (19–153)	0.17
Post-operative bleeding (CC)	285.25 ± 65.6	302.05 ± 68.76	0.65

*BT* Bleeding Time; *S* Second; *CC* Milliliter

**Table 3:** Effect of administration of ketorolac and Paracetamol on hemodynamic variables

parameter	Paracetamol (***n*** = 30)	Ketorolac (***n*** = 30)	***P***-value
MAP	87.742 (11.026)	86.451 (8.958)	0.59
HR	86.2 ± 7.2	85.5 ± 11.7	0.44
SpO2	98.4 ± 1.4	98.4 ± 1.3	0.8

*HR* Heart rate; *MAP* Mean arterial blood pressure; *SpO2* Arterial oxygen saturation

Weaning time significantly lower in the Paracetamol group than the ketorolac group (*p* = 0.003). (Table 4).

**Table 4:** Effect of administration of ketorolac and Paracetamol on outcomes

Parameter	Paracetamol (***n*** = 30)	Ketorolac (***n*** = 30)	***P***-value
WT,(hr.)	11.76 ± 1.21	18.56 ± 1.14	0.003^a^
MI, n (%)	0 (0)	0 (0)	–
Hospital mortality, n (%)	0 (0)	0 (0)	–
CVA, n(%)	0 (0)	0 (0)	–
TIA, n(%)	0 (0)	0 (0)	–
Postoperative Cr (mg/dl)	0.99 ± 0.12	1.01 ± 0.16	0.81

*hr* Hour;*n* Number; *WT* Weaning time;*MI* Myocardial infarction; *CVA* Cerebral vascular accident *TIA* Transient ischemic attack *SD* Standard deviation; *Cr* Serum-creatinine; a means significant

Comparison of the two groups receiving ketorolac and Paracetamol showed that there was no significant difference between the two groups in terms of the hospital mortality rate MI, CVA, TIA, and postoperative serum creatinine(*P* > 0.05) Table 4. There were no differences between-group in renal function tests.

## Discussion

Control of pain management in the postoperative care setting is of the greatest importance for patients who experienced CABG. Therefore, pharmacological and interventional approaches have been developed for postoperative analgesia. Currently, there is an increase in the mean age of the patients and the number of comorbidities in patients undergoing CABG. Overall, a method of postoperative analgesia that is cost-effective and comfortable for the patient with minimum complication rates and side effects which also shortens the duration of postoperative stay should be chosen. However, postoperative pain managing is often incomplete by the side effects of opioids; especially when used alone in large doses for an extended period, opioids can lead to acute tolerance and, more seriously, respiratory depression and hypotension. For these explanations, multimodal methods that add non-opioid agents to opioid-based regimens are promising. This study aimed to compare the effects of Ketorolac and Paracetamol on postoperative pain management. The main finding in the present study was that ketorolac more effective than Paracetamol to manage postoperative pain patients undergoing CABG surgery. Also, it can reduce postoperative additional analgesic requirements in comparison to Paracetamol with no additional adverse effects. This finding was similar to Amini S et al. study (20). Of course, their study was in congenital cardiac patients.

NSAIDs block the synthesis of prostaglandins through the inhibition of COX-1 and COX-2, thus lowering the production of acute inflammatory response mediators. By decreasing the inflammatory response to surgical trauma, NSAIDs reduce peripheral nociception. NSAIDs also appear to have a central analgesic mechanism, possibly through the inhibition of prostaglandin synthesis within the spinal cord. In general, NSAIDs have a low side-effect profile when administered for the short-term purpose of perioperative analgesia after cardiac surgery [21, 22].

Ketorolac is effective at reducing pain, and several studies have reported its safety and efficacy in the perioperative period. In many reports, the use of ketorolac as an adjuvant to a PCA opioid resulted in an opioid-sparing effect ranging from 16 to 33% [23]. The hypothesis by which ketorolac exerts these possible beneficial effects is proposed to be related to its COX-1 selectivity and minimal inhibition of COX-2 [24]. As previously discussed, the boxed warning for NSAIDs arose from specific data for the COX-2 selective NSAID, celecoxib [25] COX-2 inhibitors selectively reduce prostacyclin synthesis with no effect on thromboxane A2. Prostacyclin is a potent inhibitor of platelet aggregation; its selective blockade by COX-2 inhibitors may upset thrombosis homeostasis and cause adverse cardiovascular events. Ketorolac, on the other hand, potently blocks platelet aggregation through thromboxane A2 inhibition [24, 26]. This may be beneficial in patients with aspirin resistance to prevent CABG graft failure. The period of this antiplatelet effect can be last up to 24 h after a distinct dose. Additionally, the antiplatelet effects of ketorolac may offset the risk of hemorrhage in postoperative patients who may be hypercoagulable following exactly off-pump CABG surgery [17].

The authors previously reported the results of a randomized trial that found that oral naproxen is effective as an adjunct for the optimization of pain control and lung recovery after CABG, without increasing the risk of postoperative complications. In contrast to naproxen, intravenous ketorolac can be provided earlier in the postoperative period before the resumption of oral intake. Ketorolac provides an analgesic effect similar to that of fentanyl, but with a lower incidence of postoperative nausea and somnolence, and leads to an earlier return of bowel function. (15) With these advantages over opioids, ketorolac administration ultimately may shorten hospital length of stay.

Paracetamol has been studied in many surgical settings such as functional endoscopic sinus surgery, cholecystectomy, hysterectomy, and orthopedic surgeries with variable favorable results [27, 28]. The direction of acetaminophen via a nasogastric tube or rectally after surgery is insufficient to accomplish an antipyretic plasma concentration (10 mg/ml); this was probably mainly because of late gastric emptying after anesthesia and surgery [13, 29] In a study conducted by Cattabriga et al., they found that, in patients undertaking cardiac surgery, intravenous paracetamol in combination with tramadol delivers effective pain control [30, 31].

Paracetamol has resulted in hypotension in critically ill patients although this effect could be explained as an allergic phenomenon [32]. The remaining prostaglandin inhibitors seem to exert less marked cardiac depressant effect; in fact, the hemodynamic safety of other NSAIDs such as diclofenac and ketorolac used at antipyretic doses and analgesic doses has been reported in several studies [30].

The hemodynamic effects of NSAIDs used for postoperative pain control in patients undergoing major vascular surgery have been reported in a few studies [33, 34]. Although exogenous administration of prostaglandins has marked hemodynamic repercussions, exogenous inhibition of prostaglandin synthesis has a little hemodynamic effect. This could reflect a balance between the reduction in synthesis of prostaglandins with vasodilator and vasoconstrictor actions, with a neutral overall effect. However, NSAIDs must be used cautiously in clinical situations in which prostaglandins have been shown to have advantageous therapeutic effects, such as circulatory insufficiency, shock, myocardial ischemia, coronary spasm, and systemic and pulmonary hypertension; also, NSAIDs may antagonize the effect of antihypertensive medication. In the present study, such patients were excluded and therefore no evaluation of hemodynamic stability when the drugs were present was made.

Our study found no association between the use of 0.5 mg/kg ketorolac and mortality, MI, or clinically important hemorrhage. These results, however, are limited by unexpected differences in the baseline characteristics of the number of on-pump CABG patients and STS risk scores. On-pump CABG means a patient placed on cardiopulmonary bypass throughout surgery [35]. The STS risk score is intended for all patients who undergo CABG surgery and helps as a prognosticator of postoperative mortality [36].

### Limitations

This study has several limitations. First; the sample size was small second; this study was single-centered. We recommended future trials with a large sample size, multi-center and long duration of follow-up.

## Conclusion

In conclusion, ketorolac and Paracetamol may be produced marked postoperative pain relief after cardiac surgery. The analgesic effects of these compounds were not associated with a clinically significant impairment in hemodynamic function and mortality, MI, or clinically significant bleeding in postoperative CABG patients. So further study with similarly coordinated groups and the larger sample size is necessary to sufficiently determine the cardiovascular risks associated with administration of IV ketorolac.

## Data Availability

All data were retrieved from the institutional database and are available from the corresponding author upon reasonable request.

## Abbreviations

CABG: Coronary artery bypass graft
VAS: Visual analog pain scale;
COX-2: Cyclooxygenase-2 inhibitors
NMDA: N-methyl-D-aspartate
Euro Score ‖: European system for cardiac operative risk evaluation
AO: Aortic occlusion
MI: Myocardial infarction
CVA: Cerebral vascular accident
TIA: Transient ischemic attack
