# Correction: Comparing the effects of ketorolac and Paracetamol on postoperative pain relief after coronary artery bypass graft surgery. a randomized clinical trial

**DOI:** 10.1186/s13019-025-03524-5

**Published:** 2025-08-02

**Authors:** Fatemeh Javaherforooshzadeh, Hasan Abdalbeygi, Farahzad Janatmakan, Behnam Gholizadeh

**Affiliations:** 1 Department of Cardiac Anesthesia, Ahvaz Anesthesiology and Pain Research Center, Ahvaz Jundishapur University of Medical Sciences, Ahvaz, Iran; 2 Department of Anesthesia, Ahvaz Anesthesiology and Pain Research Center, Ahvaz Jundishapur University of Medical Sciences, Ahvaz, Iran; 3 Atherosclerosis Research Center, Ahvaz Jundishapur University of Medical Sciences, Ahvaz, Iran

**Journal of Cardiothoracic Surgery (2020) 15:80**

10.1186/s13019-020-01125-y

In this article [1], the graphics relating to Figs. 2 and 3 captions had been interchanged; The figure(s) should have appeared as shown below.

**Fig. 2:**
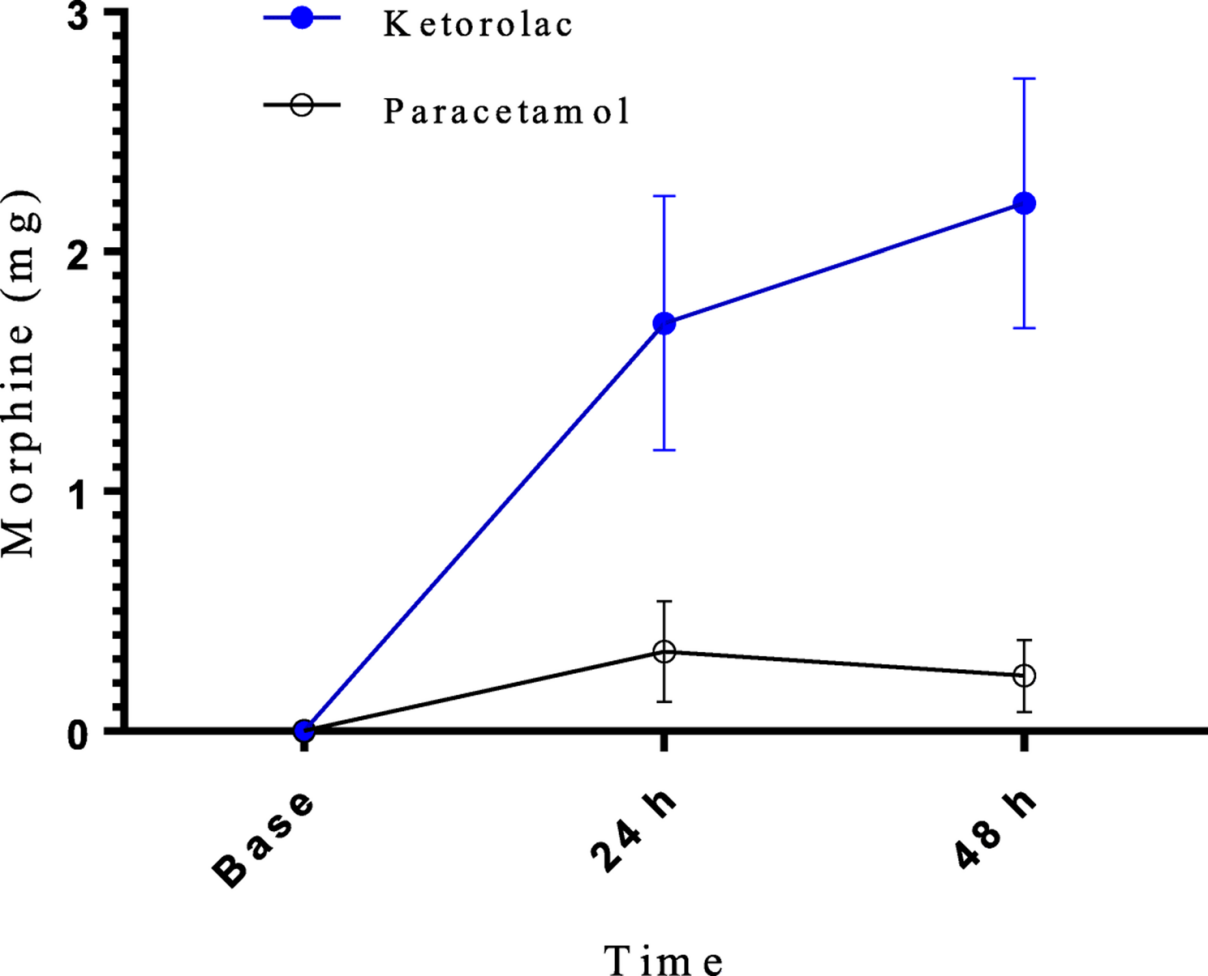
Postoperative morphine administration

**Fig. 3:**
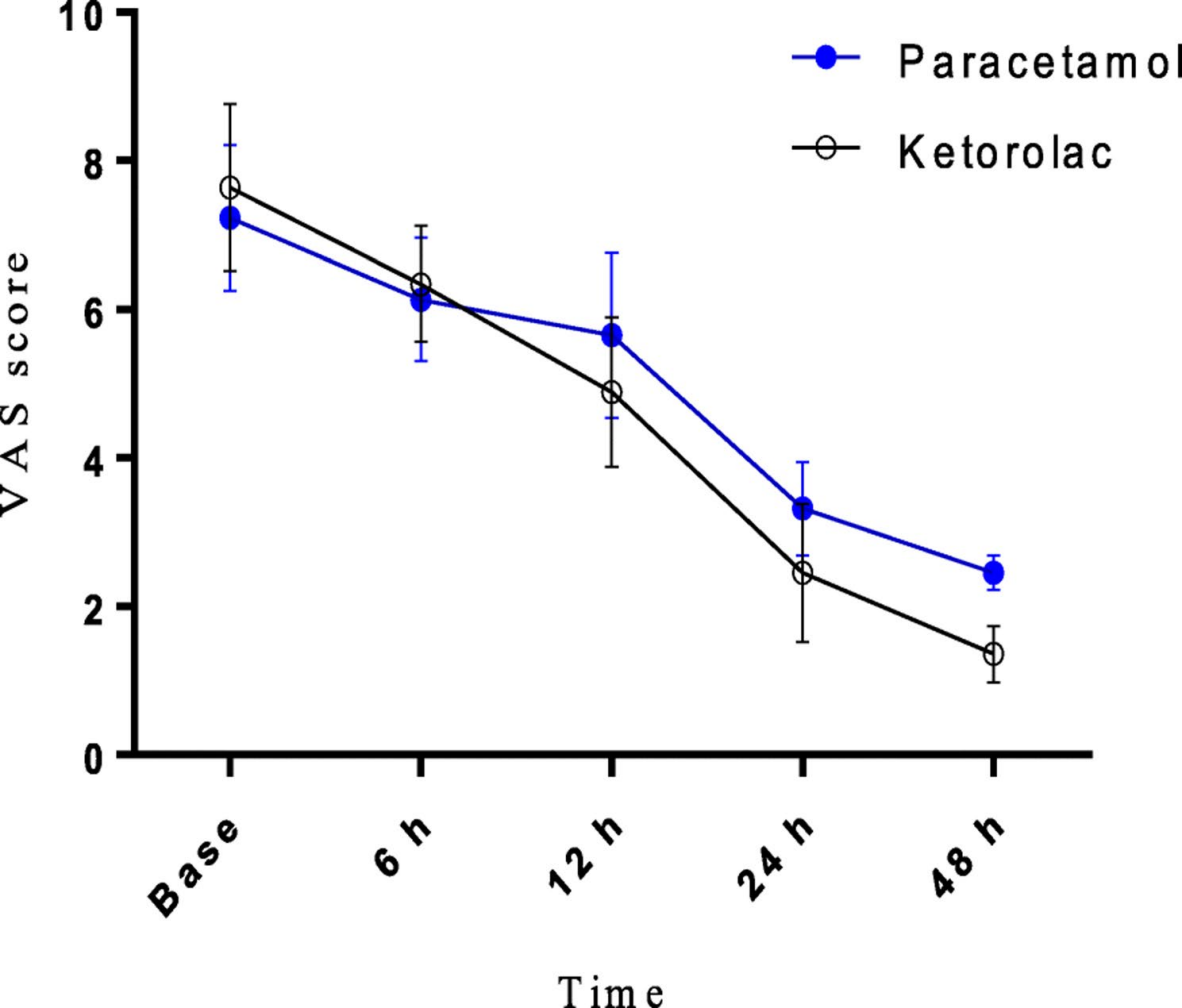
VAS score after the operation. There were significant VAS score declines in both groups (*P* < 0.05)
